# 551. Remdesivir and Tocilizumab for the Treatment of Severe COVID-19 in a Community Hospital: A Retrospective Cohort Study

**DOI:** 10.1093/ofid/ofab466.750

**Published:** 2021-12-04

**Authors:** Guillermo Rodriguez-Nava, Goar Egoryan, Daniela Patricia Trelles-Garcia, Maria Adriana Yanez-Bello, Qishuo Zhang, Chul Won Chung, Emre E Ozcekirdek, Ece Ozen, Bidhya Poudel, Heather Cohen, Harvey Friedman

**Affiliations:** 1 AMITA Health Saint Francis Hospital, Evanston, IL; 2 AMITA Health Saint Joseph Hospital, Evanston, IL

## Abstract

**Background:**

Growing evidence supports the use of remdesivir and tocilizumab for the treatment of hospitalized patients with severe COVID-19. The purpose of this study was to evaluate the use of remdesivir and tocilizumab for the treatment of severe COVID-19 in a community hospital setting.

**Methods:**

We used a de-identified dataset of hospitalized adults with severe COVID-19 according to the National Institutes of Health definition (SpO2 < 94% on room air, a PaO2/FiO2 < 300 mm Hg, respiratory frequency > 30/min, or lung infiltrates > 50%) admitted to our community hospital located in Evanston Illinois, between March 1, 2020, and March 1, 2021. We performed a Cox proportional hazards regression model to examine the relationship between the use of remdesivir and tocilizumab and inpatient mortality. To minimize confounders, we adjusted for age, qSOFA score, noninvasive positive-pressure ventilation, invasive mechanical ventilation, and steroids, forcing these variables into the model. We implemented a sensitivity analysis calculating the E-value (with the lower confidence limit) for the obtained point estimates to assess the potential effect of unmeasured confounding.

Figure 1. Kaplan–Meier survival curves for in-hospital death among patients treated with and without steroids

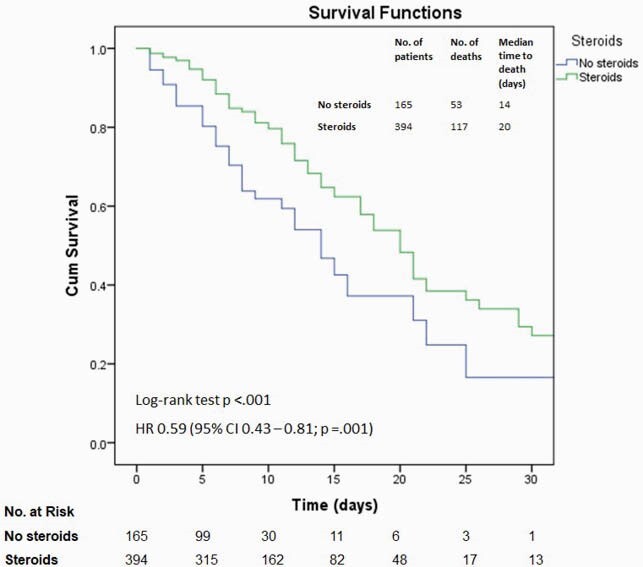

The hazard ratio was derived from a bivariable Cox regression model. The survival curves were compared with a log-rank test, where a two-sided P value of less than 0.05 was considered statistically significant.

Figure 2. Kaplan–Meier survival curves for in-hospital death among patients treated with and without remdesivir

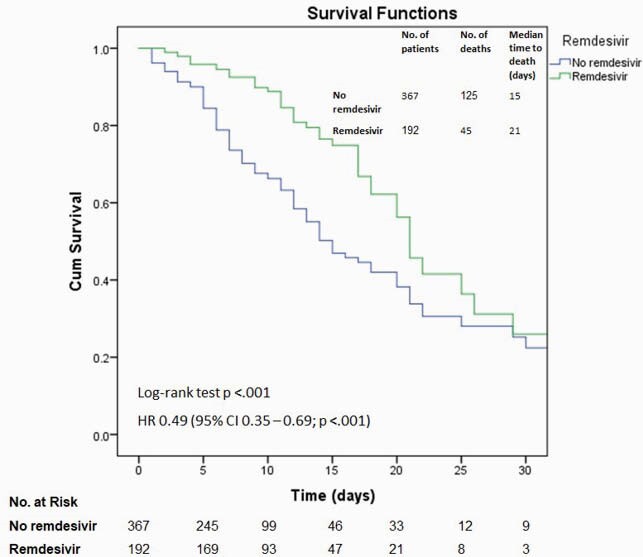

The hazard ratio was derived from a bivariable Cox regression model. The survival curves were compared with a log-rank test, where a two-sided P value of less than 0.05 was considered statistically significant.

**Results:**

A total of 549 patients were included. The median age was 69 years (interquartile range, 59 – 80 years), 333 (59.6%) were male, 231 were White (41.3%), and 235 (42%) were admitted from long-term care facilities. 394 (70.5%) received steroids, 192 (34.3%) received remdesivir, and 49 (8.8%) received tocilizumab. By the cutoff date for data analysis, 389 (69.6%) patients survived, and 170 (30.4%) had died. The bivariable Cox regression models showed decreased hazard of in-hospital death associated with the administration of steroids (Figure 1), remdesivir (Figure 2), and tocilizumab (Figure 3). This association persisted in the multivariable Cox regression controlling for other predictors (Figure 4). The E value for the multivariable Cox regression point estimates and the lower confidence intervals are shown in Table 1.

Figure 3. Kaplan–Meier survival curves for in-hospital death among patients treated with and without tocilizumab

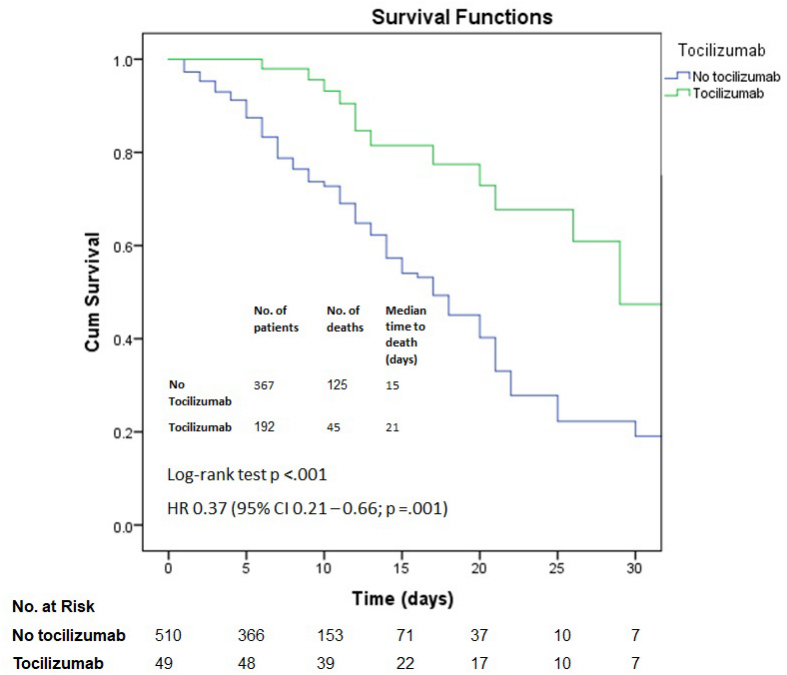

The hazard ratio was derived from a bivariable Cox regression model. The survival curves were compared with a log-rank test, where a two-sided P value of less than 0.05 was considered statistically significant.

Figure 4. Forest plot on effect estimates and confidence intervals for treatments

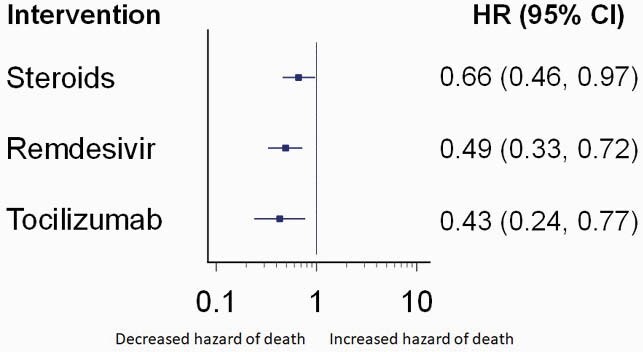

The hazard ratios were derived from a multivariable Cox regression model adjusting for age as a continuous variable, qSOFA score, noninvasive positive-pressure ventilation, and invasive mechanical ventilation.

Table 1. Sensitivity analysis of unmeasured confounding using E-values

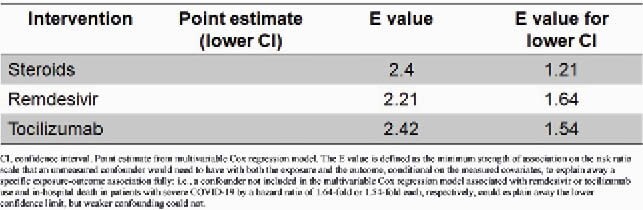

CI, confidence interval. Point estimate from multivariable Cox regression model. The E value is defined as the minimum strength of association on the risk ratio scale that an unmeasured confounder would need to have with both the exposure and the outcome, conditional on the measured covariates, to explain away a specific exposure-outcome association fully: i.e., a confounder not included in the multivariable Cox regression model associated with remdesivir or tocilizumab use and in-hospital death in patients with severe COVID-19 by a hazard ratio of 1.64-fold or 1.54-fold each, respectively, could explain away the lower confidence limit, but weaker confounding could not.

**Conclusion:**

For patients with severe COVID-19 admitted to our community hospital, the use of steroids, remdesivir, and tocilizumab were significantly associated with a slower progression to in-hospital death while controlling for other predictors included in the models.

**Disclosures:**

**All Authors**: No reported disclosures

